# Acute exercise increases fibroblast growth factor 21 in metabolic organs and circulation

**DOI:** 10.14814/phy2.12828

**Published:** 2016-06-22

**Authors:** Yuko Tanimura, Wataru Aoi, Yoshikazu Takanami, Yukari Kawai, Katsura Mizushima, Yuji Naito, Toshikazu Yoshikawa

**Affiliations:** ^1^ Department of Molecular Gastroenterology and HepatologyKyoto Prefectural University of MedicineKyotoJapan; ^2^ Faculty of humanAichi‐toho UniversityNagoyaJapan; ^3^ Laboratory of Health ScienceGraduate School of Life and Environmental SciencesKyoto Prefectural UniversityKyotoJapan; ^4^ Department of Home EconomicsOtsuma Women's UniversityTokyoJapan; ^5^ Louis Pasteur Center for Medical ResearchKyotoJapan

**Keywords:** Fibroblast growth factor 21, glucose uptake, muscle contraction, phosphorylation of Akt

## Abstract

Fibroblast growth factor 21, a metabolic regulator, plays roles in lipolysis and glucose uptake in adipose tissues and skeletal muscles. Its expression in skeletal muscle is upregulated upon activation of the phosphatidylinositol 3‐kinase/Akt signaling pathway, which is induced by exercise and muscle contraction. We examined the increase of fibroblast growth factor 21 after acute exercise in metabolic organs, especially skeletal muscles and circulation. Participants exercised on bicycle ergometers for 60 min at 75% of their $\dot{V}$O_2_max. Venous blood samples were taken before exercise and immediately after exercise. In an animal study, male ICR mice were divided into sedentary and exercise groups. Mice in the exercise group performed treadmill exercises at 30 m min^−1^ for 60 min. Shortly thereafter, blood, liver, and skeletal muscle samples were taken from mice. Acute exercise induced the increase of serum fibroblast growth factor 21 in both humans and mice, and increased fibroblast growth factor 21 expression in the skeletal muscles and the liver of mice. Acute exercise activated Akt in mice skeletal muscle. Acute exercise increases fibroblast growth factor 21 concentrations in both serum and metabolic organs. Moreover, results show that acute exercise increased the expression of fibroblast growth factor 21 in skeletal muscle, accompanied by the phosphorylation of Akt in mice.

## Introduction

Fibroblast growth factor 21 (FGF21), a metabolic regulator, produces in various tissues (Tacer et al. 2010), controls glucose metabolism and lipid metabolism (Kharitonenkov and Shanafelt 2008). Inagaki et al. (2007) reported that FGF21 can stimulate lipolysis in murine adipocytes. It regulates insulin‐independent glucose uptake in 3T3‐L1 cells that is mediated by FGF21‐dependent increase in glucose transporter (GLUT) 1 protein expression (Kharitonenkov et al. 2005). In the liver, FGF21 stimulates fatty acid utilization for energy and ketone body production (Inagaki et al. 2007). In human myotubes, FGF21 exposure increases basal and insulin‐stimulated glucose uptake in the absence (coincident with increased GLUT1) or the presence of insulin (coincident with increased GLUT1 and GLUT4) (Mashili et al. 2011). Additionally, FGF21 prevents palmitate‐induced insulin resistance in human skeletal muscle myotubes (Lee et al. 2012). These observations suggest that FGF21 plays a role in lipolysis in both the adipocytes and liver, and that it plays a role in glucose uptake in both the adipocytes and skeletal muscles.

Recently, Cuevas‐Ramos et al. (2012) reported that serum FGF21 increased significantly in women after 2 weeks of exercise. In addition, Kim et al. (2013) demonstrated that acute exercise engenders an increase in serum FGF21 concentration, which is likely to be attributable to increased liver FGF21. However, the increase of muscle FGF21 can also affect circulating FGF21. In fact, the stimulation of FGF21 expression by insulin in human skeletal muscle induces an increase in circulating FGF21 concentrations (Hojman et al. 2009). Moreover, skeletal muscle‐specific Akt1 transgenic mice exhibited increased serum FGF21 and increased skeletal muscle‐specific expression of FGF21 (Izumiya et al. 2008). These results suggest that FGF21 in skeletal muscle can regulate FGF21 in circulation.

FGF21 is increased by the activation of Akt in skeletal muscle. Izumiya et al. (2008) reported that FGF21 protein expression is upregulated by insulin, and that it is inhibited by phosphatidylinositol 3‐kinase (PI3K) inhibitor in cultured C2C12 myocytes. Additionally, skeletal muscle FGF21 mRNA and protein levels in muscle‐specific inducible Akt1 transgenic mice were higher than those in control mice (Izumiya et al. 2008). Hojman et al. (2009) reported that muscular FGF21 expression increased significantly after a few hours of insulin infusion during a hyperinsulinemic–euglycemic clamp. Therefore, FGF21 is believed to be upregulated upon activation of the PI3K/Akt signaling pathway in skeletal muscle. Two stimuli activating Akt are in skeletal muscle: insulin and contraction of skeletal muscles. Exercise decreases insulin levels, but it requires skeletal muscle contraction. Thereby, exercise is a valuable experimental tool for the assessment of insulin‐independent mechanisms that activate Akt. Several reports have described that acute exercise (treadmill exercise) acutely activated the PI3K/Akt signaling pathway in both human and animal skeletal muscle (Sakamoto et al. 2003, 2004; Deshmukh et al. 2006). Additionally, results of several studies have demonstrated that the mechanical stretch in C2C12 myotube activates Akt (Zhan et al. 2007; Chambers et al. 2009). Therefore, we examined whether acute exercise increases FGF21 in serum and metabolic organs, especially skeletal muscle associated with the activation of Akt.

## Material and Methods

### Participants

This study examined 19 healthy sedentary men [mean (SD) aged 23.7 (2.3) years old]. Their physical characteristics are presented in Table 1. No participant reported a history of smoking. None were taking any medication. We instructed participants to refrain from alcohol intake and intense physical activity on the day before the test and the test day. On experimental days, all participants arrived at the laboratory at either 0630 or 0730 h after an overnight fast. Blood samples were obtained immediately before exercise from all participants at 0700–0800 h.

**Table 1:** Characteristics of participants

*n*	19
Age (year)	23.7 ± 2.3
Height (cm)	171.7 ± 5.7
Body weight (kg)	66.0 ± 6.9
Body Mass Index (kg•m^−2^)	22.4 ± 2.0
% fat (%)	15.9 ± 3.7
Fat mass (kg)	10.7 ± 3.3
$\dot{V}$O_2_max (mL•kg^−1^•min^−1^)	42.6 ± 4.2

Values are means ± SD.

This study, which was approved by the Ethics Committee of the Kyoto Prefectural University of Medicine, conformed to the principles outlined in the Declaration of Helsinki and the ACSM Guidelines for the Use of Human Subjects. All participants were informed of the risks and procedures related to the experiment before their informed written consent to participate was obtained.

### Exercise protocol

Participants were examined for maximal oxygen uptake ($\dot{V}$O_2_max). Subsequently, they were administered a submaximal cycle exercise test. The tests were administered at least 4 days apart, within 1 month of the $\dot{V}$O_2_max test. Then $\dot{V}$O_2_max was ascertained using an incremental bicycle exercise test to exhaustion. The breath‐by‐breath oxygen uptake and carbon dioxide production were monitored using a cycle ergometer (AE280S; Minato Medical Science Co. Ltd., Osaka, Japan). This protocol comprised 2 min of unloaded pedaling and subsequent incremental exercise. The workload was increased to 60, 80, and 100 W for 2 min each. Subsequently, the workloads increased by 30 W every 3 min until exhaustion. Objective criteria for maximal effort included at least two of the following: (1) increased workload without a corresponding increase in $\dot{V}$O_2_; (2) respiratory exchange quotient equal to or greater than 1.10; (3) a pedal cadence lower than 50 rpm in spite of maximal voluntary effort. The highest O_2_ uptake over a 30‐sec period was defined as $\dot{V}$O_2_max. After at least a 12 h overnight fast, in the submaximal cycle exercise test, participants exercised on a bicycle ergometer for 60 min at 75% $\dot{V}$O_2_max after resting supine for 30 min.

### Blood analysis

Blood was sampled from the antecubital vein in the arm before and immediately after exercise. These samples were centrifuged immediately at 1,200 *g* for 10 min at 4°C. After separation, serum was dispensed into a plain microtube and was stored at −20°C for later analysis.

Circulating FGF21 was measured in serum using a commercially available enzyme‐linked immunosorbent assay (ELISA; BioVendor Laboratory Medicine Inc., Modrice, Czech Republic) following the manufacturer's instructions. Serum adrenaline concentration was measured using a human epinephrine/adrenalin ELISA kit (Cusabio Biotech Co. Ltd., Hubei, China). Serum glycerol concentration was measured using a colorimetric kit (Cayman Chemical, Ann Arbor, MI). Serum concentrations of insulin, glucose, nonesterified fatty acid (NEFA), and 3‐hydroxybutyric acid (3‐HB) were determined using enzymatic and colorimetric methods (Falco Biosystems Ltd., Kyoto, Japan). These serum concentrations were adjusted according to changes in serum volume using the Dill and Costill equation (Dill and Costill 1974).

### Animals and exercise design

This study complied with the principles and guidelines of the Japanese Council on Animal Care. It was also approved by the Committee for Animal Research of Kyoto Prefectural University of Medicine. Five‐week‐old male ICR mice were obtained from Shimizu Laboratory Supplies Co., Ltd. (Kyoto, Japan) and were acclimatized to an air‐conditioned (22 ± 2°C) room with a 12 h light–dark cycle (lights on from 0730 h to 1930 h) for 1 week. During the first 2 weeks, the level of exercise was increased gradually from running for 15 min with a treadmill set at a speed of 10 m min^−1^ to 30 m min^−1^ (three times per week). The mice were divided into two groups: a sedentary control group (CON, *n *=* *8) and a running group (RUN, *n *=* *10). On experimental days, after overnight fasting, the mice ran on the treadmill at an initial speed of 30 m min^−1^ for 60 min, with the exception of the CON group. These animals were killed immediately after exercise. The gastrocnemius, the liver, and the blood were removed. The tissues were frozen rapidly in −80°C.

### Tissue protein and serum analysis

These tissues were homogenized in CellLytic (Sigma‐Aldrich Corp., Tokyo, Japan). Supernatant was collected after centrifugation. Protein concentrations were quantified using a bicinchoninic acid (BCA) protein assay kit (Thermo Fisher Scientific Inc., MA). Concentration of FGF21 in the serum and the tissues was measured using a mouse FGF21 immunoassay kit (R&D Systems, MN).

Plasma glucose concentration was measured using the glucose dehydrogenase method (glutest PRO R; Sanwa Kagaku Kenkyusho Co. Ltd., Aichi, Japan). Plasma insulin, glycerol, NEFA, and 3‐HB concentrations were measured using insulin ELISA kit (Mercodia AB, Uppsala, Sweden), glycerol assay kit (Cayman Chemical, Ann Arbor, MI), NEFA‐C kit (Wako Pure Chemical Industries Ltd., Osaka, Japan), and 3‐HB kit (Sanwa Kagaku Kenkyusho Co., Ltd., Aichi, Japan).

### PCR analysis

Total RNA was extracted from the mouse gastrocnemius muscles and the liver using isogen (Wako Pure Chemical Industries Ltd.), according to the manufacturer's instructions. Reverse transcription (RT) polymerase chain reaction (PCR) was performed using total RNA samples obtained from the gastrocnemius and the liver using an ABI 7300 system (Applied Biosystems). Real‐time PCR using the DNA‐binding dye SYBR Green was used for detection of the PCR products for RNA sample obtained from cells after synthesized cDNA. The following PCR primers (Sigma‐Aldrich Japan K.K., Hokkaido, Japan) were used: FGF21, 5′‐CAGGGAGGATGGAACAGTGGTA‐3′ (forward) and 5′‐TGACACCCAGGATTTGAATGAC‐3′(reverse); *β*‐actin, 5′‐TATCCACCTTCCAGCAGATG‐3′ (forward) and 5′‐AGCTCAGTAACAGTCCGCCTA‐3′ (reverse). The respective ratios of the other signals to that of *β*‐actin were calculated for all samples. Normalized values were expressed as a relative value using the value of the RUN group as 1.

### Western blotting

Protein lysate obtained from the gastrocnemius muscles was separated using 10% sodium dodecyl sulfate polyacrylamide gel electrophoresis (SDS–PAGE) and was transferred to nitrocellulose membranes. The blots were incubated with primary antibody against Akt and p‐Akt (#9272 and #9271; Cell Signaling Technology Inc., MA) and secondary antibody (anti‐rabbit IgG, POD–linked species‐specific whole antibody; GE Healthcare Bio‐Sciences, Buckinghamshire, UK). Then, the target protein was visualized using enhanced chemiluminescence (ECL plus; GE Healthcare Bio‐Sciences, Piscataway, NJ). Band densities were analyzed using software (Multi Gauge ver. 3.11; Fujifilm Medical Co. Ltd., Tokyo, Japan).

### Statistical analysis

Normality of distribution was checked using the Shapiro–Wilk test. Data were analyzed using software (Prism 5 ver. 5.04 for Windows; GraphPad Software Inc., La Jolla, CA). In the human study, all parameters were analyzed using paired *t*‐tests. In the animal study, an unpaired *t‐*tests were used to compare physiologic responses measured between CON and RUN groups. Statistical significance was inferred at the 5% level. Results are presented as means ± SD.

## Results

### Elevation of serum FGF21 in response to acute exercise in humans and mice

**Figure 1:**
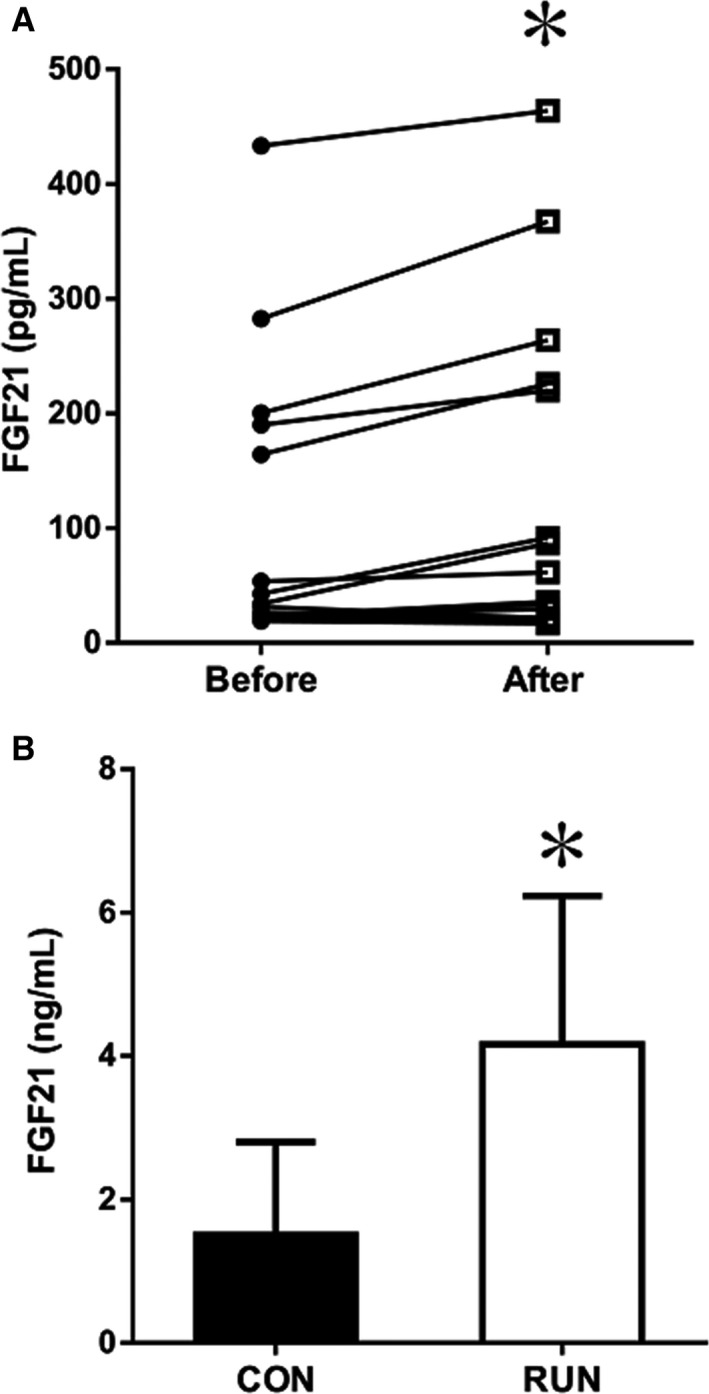
Exercise increases serum FGF21 (A) in humans (*n *=* *19) and (B) in mice (CON,* n *=* *8; RUN,* n *=* *10). Values are means ± SD. *P <* *0.05 versus before or CON.

Acute exercise induced the increase of serum FGF21 in both humans and mice. We assessed serum FGF21 in 19 male participants before and after exercise as more than 7 pg mL^−1^, which is the limit of detection of ELISA kit used for this study. Acute exercise at 75% $\dot{V}$O_2_max for 60 min increased serum FGF21 in healthy men (Fig. 1A). In mice, the serum FGF21 in the RUN group was significantly higher than that in the CON group (Fig. 1B).

### Change of lipid and glucose metabolic parameters in response to acute exercise in both humans and mice

Acute exercise increased the lipolysis parameters, the NEFA and the 3‐HB in healthy men (Table 2). Similar to the results found for humans, the serum 3‐HB lipolysis parameter increased in the RUN group in mice (Table 3). The glucose metabolic parameters, insulin and glucose, did not change by acute exercise in human (Table 2). However, the serum glucose decreased in the RUN group in mice (Table 3).

**Table 2:** Metabolic parameters in humans

	Before	After
*n*	19	
Adrenaline (pg•mL^−1^)	74.8 ± 36.7	125.6 ± 107.3
Insulin (*μ*U•mL^−1^)	4.4 ± 2.2	3.8 ± 2.9
Glucose (mg•dL^−1^)	88.7 ± 5.8	91.8 ± 11.8
NEFA (mEq•L^−1^)	0.6 ± 0.3	1.4 ± 0.7^a^
3‐hydroxybutyric acid (μmol L^−1^)	36.2 ± 24.6	81.7 ± 42.4^a^

Values are means ± SD.

NEFA, nonesterified fatty acid.

a *P *<* *0.05 versus before.

**Table 3:** Metabolic parameters in mice

	CON	RUN
*n*	8	10
Weight (g)	36.1 ± 2.6	34.9 ± 2.8
Insulin (*μ*g•L^−1^)	0.31 ± 0.16	0.35 ± 0.33
Glucose (mg•dL^−1^)	69.0 ± 7.9	43.3 ± 35.8^a^
NEFA (mEq•L^−1^)	2.23 ± 0.38	2.06 ± 0.18
3‐hydroxybutyric acid (μmol L^−1^)	85.0 ± 27.1	119.3 ± 38.0^a^

Values are means ± SD.

NEFA, nonesterified fatty acid.

a *P *<* *0.05 versus CON.

### Elevation of muscle FGF21 and Akt phosphorylation in response to acute exercise in mice

**Figure 2:**
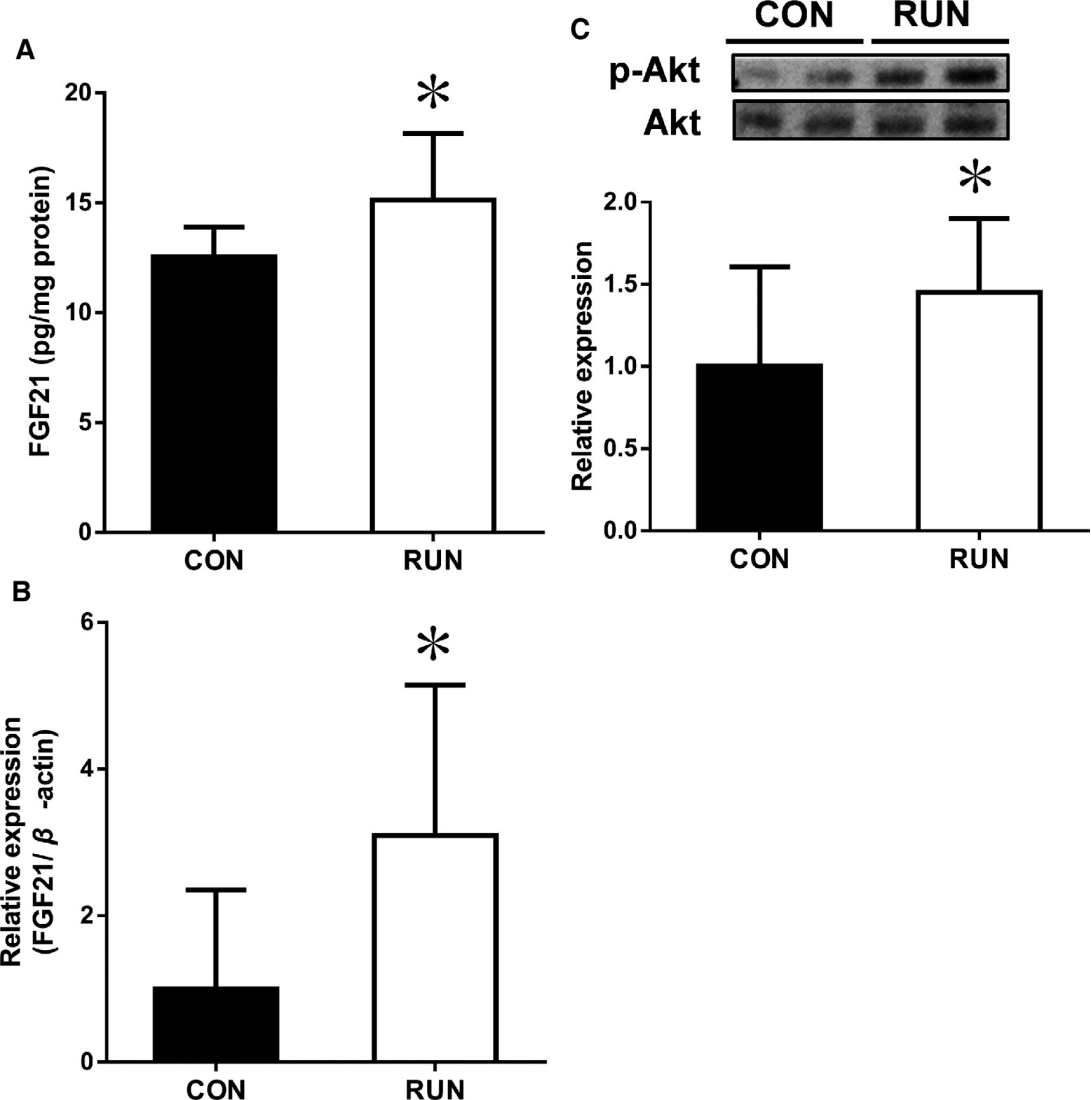
Exercise increases (A) FGF21 protein, (B) FGF21 mRNA, and (C) p‐Akt/Akt protein of skeletal muscle in mice (CON,* n *=* *8; RUN,* n *=* *10). Values are means ± SD. *P <* *0.05 versus CON.

Acute exercise increased FGF21 in the skeletal muscle. This increase established both protein and mRNA levels (Fig. 2A and B). We investigated whether the increase of FGF21 in the skeletal muscle results from the activation of Akt. Phosphorylation of Akt was increased in the RUN group compared with the CON group, but the total Akt was not changed between groups (Fig. 2C).

### Elevation of liver FGF21 in response to acute exercise in mice

**Figure 3:**
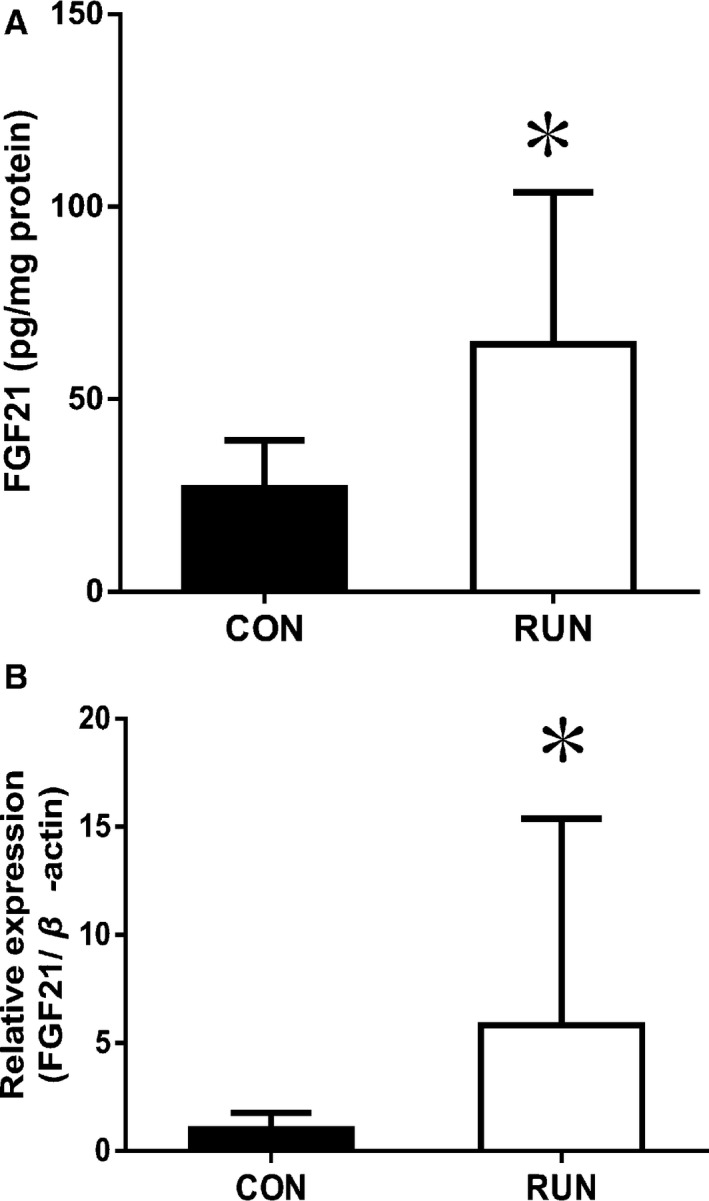
Exercise increases (A) FGF21 protein and (B) FGF21 mRNA of liver in mice (CON,* n *=* *8; RUN,* n *=* *10). Values are means ± SD. *P <* *0.05 versus CON.

Actually, FGF21 (protein level and mRNA) of the liver in the RUN group increased significantly more than in the CON group (Fig. 3).

## Discussion

We examined whether acute exercise increases FGF21 in serum and metabolic organs, especially skeletal muscle. Our results demonstrated that acute exercise increased serum FGF21 in both humans and mice. In addition, FGF21 in mice skeletal muscle was increased along with enhanced phosphorylation of Akt by acute exercise. Although we did not obtain direct data to establish that the increase of serum FGF21 is induced by increased skeletal muscle FGF21, results suggested that skeletal muscle plays a role in the level of circulating FGF21 in mice.

Several studies have examined the effects of acute exercise on circulating FGF21 in human (Cuevas‐Ramos et al. 2012; Kim et al. 2013; Hansen et al. 2015; Slusher et al. 2015). Serum FGF21 concentration in human is the difference of individuals. Hojman et al. (2009) suggested that insulin induces an increase in circulating FGF21 but showed that plasma FGF21 could only be detectable in four of 14 subjects at rest. Thereby, insulin sensitivity may be related to FGF21 in serum at rest. Actually, there's a lot of individual variation in serum FGF21 in our study. In contrast to our finding, Cuevas‐Ramos et al. (2012) reported that no increase of serum FGF21 was found after intense exercise in young women. This difference between our results and those found by Cuevas‐Ramos et al. (2012) might result from differences in the sex of participants. Hojman et al. (2009) reported that positive correlation between muscle FGF21 and insulin was found only for men, not for women. Estrogen stimulates Akt phosphorylation (Rogers et al. 2009), which may affect the elevation of FGF21 via Akt response to exercise in women. In fact, Cuevas‐Ramos et al. (2012) did not control for their women participants’ menstrual cycle status. Therefore, the concentrations of FGF21 in their participants might remain unchanged. A report of a study by Kim et al. (2013) reported the elevation of serum FGF21 after acute exercise, corresponding with our data. They found that acute exercise increases serum FGF21 concentration together with enhanced lipolysis. Their suggestion agrees with our results of lipolysis parameters.

Exercise is well known to engender metabolic improvement. Skeletal muscle is the most important organ in terms of energy metabolism, although details of the mechanism remain unclear. Results show the increase of FGF21 along with the activation of Akt in skeletal muscle after acute exercise in mice. Consequently, this increase of FGF21 in skeletal muscle is likely to be induced by activated Akt signaling, which is supported by the results of a previous study (Izumiya et al. 2008) showing that skeletal muscle FGF21 concentrations are high in skeletal muscle‐specific Akt1 transgenic mice. Insulin stimulation is the representative factor that activates Akt in skeletal muscle. In fact, insulin increased the FGF21 in skeletal muscle (Hojman et al. 2009) and C2C12 (Izumiya et al. 2008) in a PI3K/Akt‐dependent manner. Our results showed unchanged serum insulin after exercise for both humans and animals. Another possibility is that muscle contraction also induced Akt activation. Sakamoto et al. (2003, 2004) reported that exercise induces an intensity‐dependent increase in the phosphorylation of Akt in skeletal muscle, which would be induced by a mechanical stress with muscle contraction. In addition, passive stretching and electrical stimulation increased phosphorylation of Akt in isolated muscle of mice (Ito et al. 2006). Therefore, we inferred that the activating Akt of skeletal muscle could induce the increase of muscle FGF21 in this study.

Little is known about tissue influences on the concentration of circulating FGF21. Dutchak et al. (2012) reported that feeding increased the expression of FGF21 in white adipose tissues (WAT), a major FGF21‐contained tissue, although it did not increase circulating FGF21, which indicates that adipose‐derived FGF21 acts in an autocrine/paracrine manner. However, skeletal muscle‐derived FGF21 also act in a paracrine/autocrine and endocrine manner. Human primary myotubes secreted FGF21 into culture medium by stimulation of ionomycin. (Besse‐Patin et al. 2014). In adeno‐myrAkt1 C2C12 myocytes, FGF21 concentration in both the cell lysate and the cell media is higher than the control (Izumiya et al. 2008). In addition, in a human experiment, serum and skeletal muscle FGF21 increased in response to acute hyperinsulinemia in healthy young men (Hojman et al. 2009). They also reported that a positive correlation exists between fasting insulin and muscle FGF21 mRNA and plasma FGF21, which suggests that muscle FGF21 increase plasma FGF21. The muscle FGF21 concentration increases rapidly when the Akt in muscle is activated by acute action in healthy young men who have low baseline concentration of muscle FGF21 which results in the elevation of circulation FGF21. Thereby, the activation of Akt induced by acute exercise (muscle contraction) might increase circulation FGF21.

Our data show that liver FGF21 also significantly increased after acute exercise. Results suggest that hepatic FGF21 can be secreted into circulation and functions as endocrine mechanisms, as shown in the previous study (Murata et al. 2011). Both fasting and the overfeeding increase the circulating FGF21 concentration, along with the concentration of liver FGF21 (Badman et al. 2007; Potthoff et al. 2009). In addition, Li et al. (2010) reported that serum FGF21 concentrations are correlated with hepatic FGF21 mRNA in nonalcoholic fatty liver disease patients. Therefore, the elevation of liver FGF21 can mediate the elevation of circulating FGF21 concentration. In the exercise condition, FGF21 secreted from liver would also affect the circulating level of FGF21 more than muscle‐secreted FGF21, as shown by Hansen et al. (2015). In contrast, muscle‐derived FGF21 may rather mainly act through paracrine and autocrine effects in muscle tissues locally, which improves whole‐body metabolism via improving metabolism of the muscle, the major metabolic organ. Moreover, various studies showed that muscle FGF21 increases with mitochondria stress conditions such as chronic high insulin (Hojman et al. 2009), lipodystrophy (Lindegaard et al. 2013), and UCP‐1 deficiency (Keipert et al. 2014), which may mean one of negative feedback systems to maintain normal metabolic function in the body, as suggested in the concept of Ji et al. (2015). In fact, Kim et al. (2013) reported that the elevation of FGF21 in skeletal muscle improves insulin resistance and reduces fat mass in autophagy‐deficient mice which shows mitochondrial dysfunction. Furthermore, Guridi et al. (2015) reported that activation of mTORC1 in muscle improves whole‐body metabolism through FGF21 production in muscle. These studies supported the concept that muscle‐derived FGF21 regulates the whole body metabolism by enhancing glucose uptake, fatty acid oxidation, and glycolysis in skeletal muscle through mainly autocrine/paracrine, secondly endocrine. Namely, exercise induced increase of FGF21 in muscle also plays a role in metabolic improvement in whole body.

Generally, during fasting, NEFA is released from adipocytes into blood and partially converted to acetyl‐CoA, which produces ketone bodies in the liver. Thereby, fasting or a ketogenic diet (similar to chronic starvation) induces FGF21 expression in mice liver via NEFA (Badman et al. 2007; Potthoff et al. 2009). The NEFA binds to activated peroxisome proliferator‐activated receptor *α* (PPAR*α*), forming a heterodimer with retinoid X receptors (RXRs) and inducing the expression of FGF21 (Mai et al. 2009). Fletcher et al. (2012) concluded that enhanced hepatic FGF21 and its primary signaling mediators by daily exercise and caloric restriction may partially explain the improvement of fatty liver. Exercise encourages lipolysis by the increase of catecholamine and the decrease of insulin. At this point, exercise is similar to fasting or starvation in terms of energy and increases NEFA in liver. In our human study described herein, participants performed exercise following 12 h of fasting. NEFA and 3‐HB increased significantly after exercise, concomitantly with the increase of FGF21. Although NEFA did not increase in the RUN group in our animal study, 3‐HB increased significantly in the RUN group than the CON group. Therefore, these increases of NEFA and/or 3‐HB might affect the increase in serum FGF21 to some degree via the production of FGF21.

In conclusion, results show that acute exercise increased the circulating FGF21 concentrations of humans and mice. Moreover, the expression of FGF21 in metabolic organs was increased by acute exercise in mice, which was accompanied by the phosphorylation of Akt in skeletal muscle. Further studies must be undertaken to clarify the effects of organ FGF21 on its increase in circulation by acute exercise.

## Conflict of Interest

None declared.
